# Retraction: The botanical drug PBI-05204, a supercritical CO^2^ extract of Nerium oleander, inhibits growth of human glioblastoma, reduces Akt/mTOR activities, and modulates GSC cell-renewal properties

**DOI:** 10.3389/fphar.2026.2021904

**Published:** 2026-10-15

**Authors:** Frontiers Editorial Office

The journal retracts the 11 September 2020 article cited above.

Following publication, concerns were raised regarding the integrity of the images in the published figures. The authors failed to provide the raw data or a satisfactory explanation during the investigation, which was conducted in accordance with Frontiers’ policies. Given the concerns about the validity of the data, and the lack of raw data, the editors no longer have confidence in the findings presented in the article.

This retraction was approved by the Chief Editors of *Frontiers in Pharmacology* and the Chief Executive Editor of Frontiers. The authors received a communication regarding the retraction and had a chance to respond. This communication has been recorded by the publisher.

Frontiers would like to thank Sholto David for contacting the journal regarding the published article.

